# Placental microRNA methylome signatures may serve as biomarkers and therapeutic targets for prenatally opioid-exposed infants with neonatal opioid withdrawal syndrome

**DOI:** 10.3389/fgene.2023.1215472

**Published:** 2023-06-15

**Authors:** Uppala Radhakrishna, Swapan K. Nath, Lavanya V. Uppala, Avinash Veerappa, Ariadna Forray, Srinivas B. Muvvala, Raghu P. Metpally, Richard C. Crist, Wade H. Berrettini, Lori M. Mausi, Sangeetha Vishweswaraiah, Ray O. Bahado-Singh

**Affiliations:** ^1^ Department of Obstetrics and Gynecology, Oakland University William Beaumont School of Medicine, Royal Oak, MI, United States; ^2^ Arthritis and Clinical Immunology Program, Oklahoma Medical Research Foundation, Oklahoma City, OK, United States; ^3^ College of Information Science and Technology, Peter Kiewit Institute, The University of Nebraska at Omaha, Omaha, NE, United States; ^4^ Department of Genetics, Cell Biology and Anatomy College of Medicine, University of Nebraska Medical Center, Omaha, NE, United States; ^5^ Department of Psychiatry, Yale School of Medicine, New Haven, CT, United States; ^6^ Department of Molecular and Functional Genomics, Danville, PA, United States; ^7^ Department of Psychiatry, University of Pennsylvania Perelman School of Medicine, Philadelphia, PA, United States; ^8^ Geisinger Clinic, Danville, PA, United States

**Keywords:** MicroRNAs, opioid use disorder (OUD), neonatal opioid withdrawal syndrome (NOWS), methylation, biomarkers

## Abstract

**Introduction:** The neonate exposed to opioids in utero faces a constellation of withdrawal symptoms postpartum commonly called neonatal opioid withdrawal syndrome (NOWS). The incidence of NOWS has increased in recent years due to the opioid epidemic. MicroRNAs (miRNAs) are small non-coding RNA molecules that play a crucial role in gene regulation. Epigenetic variations in microRNAs (miRNAs) and their impact on addiction-related processes is a rapidly evolving area of research.

**Methods:** The Illumina Infinium Methylation EPIC BeadChip was used to analyze DNA methylation levels of miRNA-encoding genes in 96 human placental tissues to identify miRNA gene methylation profiles as-sociated with NOWS: 32 from mothers whose prenatally opioid-exposed infants required pharmacologic management for NOWS, 32 from mothers whose prenatally opioid-exposed infants did not require treat-ment for NOWS, and 32 unexposed controls.

**Results:** The study identified 46 significantly differentially methylated (FDR *p*-value ≤ 0.05) CpGs associated with 47 unique miRNAs, with a receiver operating characteristic (ROC) area under the curve (AUC) ≥0.75 including 28 hypomethylated and 18 hypermethylated CpGs as potentially associated with NOWS. These dysregulated microRNA methylation patterns may be a contributing factor to NOWS pathogenesis.

**Conclusion:** This is the first study to analyze miRNA methylation profiles in NOWS infants and illustrates the unique role miRNAs might have in diagnosing and treating the disease. Furthermore, these data may provide a step toward feasible precision medicine for NOWS babies as well.

## Introduction

Opioid use disorder (OUD) is a global health crisis that has led to a sharp increase in drug overdose deaths. Regular use of opioids during pregnancy can lead to Neonatal Opioid Withdrawal Syndrome (NOWS). This illness is associated with increased morbidity and mortality in infancy and is a significant risk factor with negative impacts on the neurodevelopment of infants. Every 15 min a baby is born to a mother with an OUD (Honein et al., 2019), and 8.7 million children in the US have a parent with OUD (Lipari and Van Horn, 2013). Many *in-utero* opioid-exposed neonates are born prematurely below 32 weeks of gestational age (Cleary et al., 2011) with a wide range of neurobiological symptoms. Acutely, neonates experiencing NOWS have autonomic nervous system dysfunction, insomnia, feeding difficulty, inconsolable crying, irritability, and seizures; they may require opioid medication and/or extended hospitalization. Longer-term sequalae of NOWS can include learning and cognitive disabilities (Baldacchino et al., 2015). The most common pharmacotherapies of choice for pregnant women with OUD include methadone or buprenorphine (Meyer et al., 2015). Previous genome-wide DNA methylation and transcriptome studies reported differentially methylated and expressed genes and multiple dysregulated biological pathways closely associated with mothers of infants with NOWS (Radhakrishna et al., 2021a; Radhakrishna et al., 2021b). Furthermore, gene-specific methylation variation profiles in mothers with OUD were reported in comparison to normal controls (Chorbov et al., 2011; Wachman et al., 2013; Wachman et al., 2018) and genetic variants were associated with NOWS risk and severity (Oei et al., 2012; Wachman et al., 2013; Wachman et al., 2015; Mactier et al., 2017; Wachman et al., 2017; Metpally et al., 2019).

Non-coding RNAs (ncRNAs) are emerging as key regulators of cellular processes such as genome integrity, cell growth, proliferation, differentiation, development, chromatin organization, gene expression, and signal transduction (Puvvula, 2019; Li et al., 2020). There are many classes of ncRNAs, with a few functionally important types including microRNA (miRNA), long non-coding RNA (lncRNA), circular RNA (circRNA), piwi-interfering RNA (piRNA), and small nucleolar RNA (snoRNAs). Dysregulation of these ncRNAs is involved in many cancers and also drug abuse (Zhang K. et al., 2016). MicroRNAs (miRNAs) are indeed one of the most prominent and extensively studied classes of non-coding RNAs (ncRNAs). They are small, single-stranded non-coding RNAs, and are highly conserved post-transcriptional negative regulators of gene expression by binding to the 3’ untranslated region of target mRNA (Bartel, 2004; Zhang et al., 2017). MicroRNAs have gained significant attention in the field of molecular biology and genetics due to their involvement in various biological processes, including gene regulation and disease pathogenesis (Contreras and Rao, 2012; Banerjee and Sen, 2015; Iacomino and Siani, 2017; Sliwinska et al., 2017; Zhou et al., 2018). Many diseases and conditions are influenced by variations in miRNA gene expression. Variations in miRNAs expression can result from deletions and duplications of larger sequences or chromosomes, mutations involving miRNA loci, or epigenetic methylation, among other factors (Ali Syeda et al., 2020).

Epigenetic mechanisms such as DNA methylation play an important role in regulating miRNA expression. Most of the miRNA genes were found in CpG-rich regions (Bianchi et al., 2017) and therefore DNA methylation may play an important role in altered miRNA expression. miRNAs can directly target epigenetic factors, such as DNA methyltransferases or histone deacetylases, thus regulating chromatin structure. These miRNAs are useful not only as diagnostic biomarkers for drug exposure but may also be neuroprotective in the context of drug use (Saugstad, 2010).

Since the 1993 discovery of miRNAs in *Caenorhabditis elegans* (Lee et al., 1993), there are now approximately 2500 defined human miRNAs, though many are without experimental validation (Chiang et al., 2010; Kozomara and Griffiths-Jones, 2014). The miRNAs do not require precise complementarity for target recognition (Christopher et al., 2016), a single miRNA can regulate the expression of multiple genes as its targets, while one gene may be targeted by many miRNAs (Adlakha and Saini, 2014). miRNA-based regulation is implicated in many disease etiologies and has been studied for treatment. Hence, miRNAs serve as an ideal unifying molecular marker to better understand the pathophysiological processes that may regulate gene expression. However, miRNAs association in placental tissues of mothers of *in-utero* opioid-exposed infants born with NOWS has not yet been fully addressed.

The placenta is a temporary fetal organ, which allows the exchange of nutrients and gases between the mother and the developing fetus. The placenta governs the development of fetal organs including the brain (Burton and Fowden, 2015). During pregnancy, miRNAs of placental origin are released continually in the maternal circulatory system, indicating that these miRNAs might serve as biomarkers for placental function during pregnancy and in cellular communication (Mouillet et al., 2015). Prior reports indicate that most addictive drugs easily cross the placenta and can affect fetal brain development (Ross et al., 2015).

As such, placenta-derived miRNAs may be assessed as surrogate markers for brain health at birth, following *in-utero* opioid exposure, and may predict the severity of NOWS before the emergence of physiological signs of withdrawal. However, the potential roles of placental miRNAs in OUD-pregnancy and NOWS outcomes are unknown. This study was undertaken to identify methylation differences in miRNA-encoding genes in the placentas of mothers with OUD who gave birth to infants with NOWS, which may help to identify potential biomarkers of NOWS.

## Materials and methods

This study was approved by the Institutional Ethics Committee of William Beaumont Health System, Royal Oak, MI, USA (2019–086) and informed consent was waived as the research utilized paraffin blocks with archived records. The study was carried out under the principles of the Declaration of Helsinki. The study participants included only European Americans in the United States and details on the study cohort have been previously published (Radhakrishna et al., 2021a; Radhakrishna et al., 2021b). Inclusion criteria were the diagnosis of infants with NOWS born to opioid-misusing mothers. Mothers were evaluated for OUD using the Diagnostic and Statistical Manual of Mental Disorders, Fifth Edition, or DSM-5, assessment criteria (Hasin et al., 2013) by physicians/psychiatrists. Newborns with NOWS were studied by neonatologists based on ICD-10 clinical criteria (ICD-10 P96.1). All infants born to mothers with OUD were monitored in the inpatient unit for 4–5 days to observe for signs of NOWS. The infant was scored using the Finnegan Neonatal Abstinence Scoring Tool (FNAST), which was used to determine the use of pharmacological management with morphine. This scoring was done by the *postpartum* nurses and/or NICU nurses.

To identify DNA methylation patterns of miRNA encoding genes and their effect on expression, we analyzed 96 Formalin-Fixed Paraffin-Embedded (FFPE) archived placental tissue specimens for which DNA methylation and RNA sequencing (RNA-seq) data was recently published by our NOWS consortium (Radhakrishna et al., 2021a; Radhakrishna et al., 2021b). Placental specimens were obtained 2 cm away from the umbilical cord insertion site on the mother’s side of the placenta and were divided into 3 groups. Group 1, consisted of 32 infants prenatally exposed to opioids who received pharmacologic treatment for NOWS symptoms (+Opioids/+NOWS), and 32 infants, prenatally exposed to opioids that did not require pharmacologic therapy for NOWS (+Opioids/-NOWS) and 32 unexposed controls. Among 64 opioid-exposed mothers, 61 (95%) had a history of tobacco smoking. Among 32 mothers (unexposed controls) consisted of individuals who did not have any opioid use and did not have infants diagnosed with NOW (-Opioids/-NOWS, control), 11 (34%) were smokers.

### DNA extraction, bisulfite conversion, and restoration

DNA was extracted from the FFPE placental blocks using a QIAamp DNA FFPE tissue kit (Qiagen, catalog no. 56404) according to the manufacturer’s protocol. Infinium HD FFPE Restore kit (Illumina, San Diego, CA) was used for DNA restoration (Siegel et al., 2014) followed by bisulfite conversion of 500 ng of DNA using the EZ DNA Methylation kit (Zymo Research, Irvine, CA) according to the manufacturer’s instructions. The methodology has been detailed earlier (Radhakrishna et al., 2016; Radhakrishna et al., 2021b).

### Illumina Infinium Methylation EPIC BeadChip

Genome-wide DNA methylation profiling was performed using the Infinium Methylation EPIC BeadChip array (Illumina Inc., San Diego, CA) with bisulfite-treated genomic DNA according to the manufacturer’s protocol (Vishweswaraiah et al., 2019; Radhakrishna et al., 2021b). The Methylation EPIC BeadChip microarray provides quantitative measurement of 853,307 CpG sites, including the methylation of 9,961 CpG site regulators of miRNA-encoding genes present in the array (Moran et al., 2016). The sample placement in each chip was randomized to avoid confounding and to achieve successful microarray experiments (Verdugo et al., 2009). During the preprocessing of the methylation data, CpG-sites annotated to X and Y chromosomes and/or containing SNPs near or within the probe sequence (within 10 bp of the CpG site), probes that lacked beta values, and probes with minor allele frequency exceeding 0.05 were excluded from the analysis (Chen et al., 2013; Liu et al., 2013; Wilhelm-Benartzi et al., 2013; Daca-Roszak et al., 2015), followed by the analysis of the beta values for the remaining CpG sites. SNPs within 10 bp of the microRNA binding site may affect microRNA and mRNA interactions and reduce binding affinity (Li et al., 2016).

### Network analysis of miRNAs

To establish associations between miRNAs and the biological processes they regulate in OUD/NOWs, we performed a gene ontology (GO) analysis. Pathway analysis was carried out using Ingenuity Pathway Analysis (IPA^®^) using differentially expressed miRNAs at FDR *p*-value <0.05. MiRNAs were removed from the analysis if they were duplicated or unrecognized by IPA. The most statistically significant miRNAs identified in the NOWS were used in canonical pathways.

## Results

The present cohort comprised 96 infants including 64 diagnosed with NOWS born to mothers with OUD and 32 unexposed controls (Table 1). We initially generated genome-wide methylation and gene expression measurements in the cohort mentioned above and published them (Radhakrishna et al., 2021a; Radhakrishna et al., 2021b). In the follow-up study, we aimed to identify miRNA genes in the proximity of CpG sites, in which modifications of the epigenetic profile are associated with NOWS. We analyzed the methylation of 9,961 CpG site regulators of miRNA-encoding genes present in the Illumina epic array.

**TABLE 1:** Demographic characteristics of the study subjects: opioid-exposed NOWS newborns that need treatment (+Opioids/+NOWS), mothers with prenatally opioid-exposed infants that did not require treatment for NOWS (+Opioids/-NOWS), and unexposed mothers with normal controls (-Opioids/-NOWS).

		Number of subjects	Maternal age in years- Mean (SD)	Gestational age at delivery in weeks– Mean (SD)	Alcohol history	Tobacco history	Birth weight (g) –Mean (SD)	NICU admission	NOWS development
(+Opioids/+NOWS) versus (+Opioids/-NOWS)	Cases	32	31 (4.7)	37.94 (3.16)	4 (12%)	30 (93%)	2800 (780.7)	32 (100%)	32 (100%)
	Controls	32	28 (4.9)	37.49 (2.96)	3 (9%)	31 (96%)	2752 (671.2)	11 (34%)	0
	*q*-value (FDR)	NA	0.9947	0.9947	0.9947	0.9947	0.9947	<0.00001	<0.00001
+Opioids/+NOWS plus +Opioids/-NOWS versus -Opioids/-NOWS	Cases	64	29.5 (5.1)	37.72 (3.07)	7 (11%)	61 (95%)	2776 (729.7)	43 (67%)	32 (50%)
	Controls	32	32.5 (4.1)	38.09 (3.37)	3 (9%)	11 (34%)	3117 (760.6)	3 (9%)	0
	*q*-value (FDR)	NA	0.8514	0.0053	0.0237	0.0643	0.0168	0.0052	0.0948
+Opioids/+NOWS versus -Opioids/-NOWS	Cases	32	31 (4.7)	37.94 (3.16)	4 (12%)	30 (93%)	2800 (780.7)	32 (100%)	32 (100%)
	Controls	32	32.5 (4.02)	38.09 (3.37)	3 (9%)	11 (34%)	3117 (760.6)	3 (9%)	0
	*q*-value (FDR)	NA	0.5747	0.821	0.2522	0.024	0.1243	<0.00001	<0.00001

We identified 47 significantly differentially methylated (FDR *p*-value ≤0.05) CpGs associated with 46 unique miRNAs, with a receiver operating characteristic (ROC) area under the curve (AUC) ≥0.75 which were used in the follow-up study. Detailed hypomethylated and hypermethylated miRNAs were identified in the placentas of infants with NOWS (Tables 2; Table 3; Table 4). Table 2 describes the miRNA dataset from +Opioids/+NOWS, which was compared against + Opioids/-NOWS. miRNAs such as miR-301, miR-573, and miR-548 among others were found significantly differentially methylated. Table 3 describe data distinguishing the combined two OUD groups from unexposed controls (+Opioids/+NOWS plus + Opioids/-NOWS *versus* -Opioids/-NOWS, control)**.** The differentially methylated miRNAs identified include miR-181, miR-34, miR-129, and miR-548. Finally, Table 4 defines + Opioids/+NOWS *versus* unexposed controls (-opioids/-NOWS), yielded the following differentially methylated miRNAs: miR-34, miR-10, miR-548, miR-518, miR-1909, and miR-1178.

**TABLE 2:** The analysis of +Opioids/+NOWS *versus* + Opioids/-NOWS. Significantly differentially methylated microRNAs based on FDR adjusted-p values < 0.05 provided with (AUC) ≥0.75 for NOWS detection. Details of corresponding CpG loci, chromosomes, and methylation status.

TargetID	miR	CHR	*p*-Val	FDR *p*-Val	% Methylation			CI			Methylation
					Cases	Control	Difference	AUC	lower	upper	
cg11210410	miR-1268A	9	3.12539E-38	2.70346E-32	74.62	64.79	9.83	0.82	0.72	0.93	Hyper
cg14929554	miR-5095	16	2.56839E-14	2.22166E-08	60.20	70.37	−10.17	0.76	0.65	0.88	Hypo
cg16769912	miR-558	2	1.85302E-13	1.60286E-07	60.52	70.36	−9.83	0.78	0.67	0.90	Hypo
cg13790797	miR-548G	3	4.61626E-11	3.99307E-05	49.01	58.67	−9.66	0.76	0.64	0.88	Hypo
cg04312413	miR-548F1	1	6.42158E-11	5.55466E-05	71.41	78.91	−7.50	0.77	0.66	0.89	Hypo
cg02656609	miR-301A	17	1.18711E-10	0.000102685	76.05	82.73	−6.68	0.81	0.70	0.92	Hypo
cg10046367	miR-548W	17	1.30176E-10	0.000112602	69.25	76.95	−7.70	0.83	0.73	0.93	Hypo
cg26481500	miR-1278	1	1.48897E-10	0.000128796	77.09	83.57	−6.48	0.78	0.67	0.89	Hypo
cg23632539	miR-5096	1	6.57986E-10	0.000569158	78.51	71.87	6.64	0.74	0.61	0.86	Hyper
cg15350946	miR-1273H	4	6.59135E-10	0.000570152	45.23	54.40	−9.18	0.73	0.61	0.85	Hypo
cg06769231	miR-2117	17	6.72269E-10	0.000581513	64.17	72.20	−8.03	0.76	0.64	0.87	Hypo
cg22395021	miR-548H3	6	7.95169E-10	0.000687821	61.28	69.57	−8.29	0.70	0.57	0.83	Hypo
cg00071872	miR-548AE2	16	9.20749E-10	0.000796448	14.56	9.27	5.29	0.72	0.60	0.85	Hyper
cg18363417	miR-573	4	2.94005E-09	0.00254314	73.90	80.45	−6.54	0.77	0.65	0.88	Hypo
cg21254731	miR-939	8	3.15349E-09	0.002727773	81.93	87.17	−5.24	0.86	0.77	0.95	Hypo
cg02790122	miR-1296	10	4.68281E-09	0.00405063	77.11	83.10	−5.99	0.71	0.58	0.84	Hypo
cg26042267	miR-3663HG	10	5.19928E-09	0.004497379	35.81	28.35	7.47	0.72	0.59	0.84	Hyper
cg25249290	miR-181A1HG	1	8.31433E-09	0.007191897	61.56	69.35	−7.79	0.73	0.61	0.86	Hypo
cg19350059	miR-548F5	13	8.80098E-09	0.007612844	18.63	13.02	5.61	0.74	0.62	0.87	Hyper
cg00224335	miR-4327	21	1.13961E-08	0.009857667	52.54	44.68	7.86	0.75	0.63	0.87	Hyper
cg17777998	miR-1183	7	1.58092E-08	0.013674961	38.38	46.76	−8.38	0.71	0.59	0.84	Hypo
cg13641189	miR-658	22	2.75754E-08	0.02385271	11.27	6.90	4.37	0.79	0.68	0.91	Hyper
cg03425468	miR-92B	1	3.25876E-08	0.028188258	11.44	7.06	4.38	0.75	0.63	0.87	Hyper
cg24289256	miR-548F3	1	4.69893E-08	0.040645767	72.95	79.16	−6.21	0.70	0.58	0.83	Hypo
cg03135982	miR-3650	5	4.94006E-08	0.042731525	76.26	82.01	−5.75	0.74	0.62	0.87	Hypo

**TABLE 3:** Analysis of (+Opioids/+NOWS), + (+Opioids/-NOWS), *versus* (-Opioids/-NOWS, control). Differentially methylated microRNAs and the corresponding methylated CpG sites with Target ID, Gene ID, chromosome location, *p*-value, FDR *p*-value, and % methylation change are given.

Target ID	miR	CHR	*p*-Val	FDR *p*-Val	% Methylation			AUC	CI		
					Cases	Control	Difference		lower	upper	Methylation
cg11479035	miR-1276	15	3.00299E-37	2.59759E-31	86.38	79.64	6.74	0.83	0.73	0.93	Hyper
cg13767940	miR-34B	11	2.67438E-17	2.31334E-11	9.13	16.99	−7.86	0.72	0.59	0.85	Hypo
cg23632539	miR-5096	1	2.20382E-13	1.9063E-07	80.63	73.11	7.51	0.82	0.72	0.92	Hyper
cg19782652	miR-125A	19	1.19367E-09	0.001032525	78.09	84.18	−6.09	0.92	0.85	0.99	Hypo
cg19069367	miR-6850	8	1.61143E-09	0.001393891	9.86	5.38	4.48	0.82	0.72	0.92	Hyper
cg21913981	miR-1178	12	2.14827E-09	0.001858257	73.86	66.75	7.10	0.79	0.68	0.90	Hyper
cg19756622	miR-518A1; miR-518E	19	7.21604E-09	0.006241877	81.17	75.24	5.93	0.84	0.74	0.94	Hyper
cg24774002	miR-1909	19	3.6236E-08	0.031344152	89.10	92.79	−3.69	0.83	0.73	0.93	Hypo
cg13477253	miR-548G	3	5.62137E-08	0.048624868	15.48	10.50	4.98	0.77	0.66	0.89	Hyper

**TABLE 4:** Analysis of (+Opioids/+NOWS), *versus* (-Opioids/-NOWS, control). Details of CpG targets significantly differentially methylated microRNAs in NOWS. Differentially methylated CpG sites with Target ID, Gene ID, chromosome location, *p*-value, FDR *p*-value, and % methylation change details are given.

CpG target	miR	CHR	*p*-value	FDR *p*-Val	% Methylation			AUC	CI		
					Cases	Control	diffreence		lower	upper	Methylation
cg20276377	miR-548G	3	1.98166E-23	1.71414E-17	15.95839	25.9444	−9.98601	0.65	0.53	0.77	Hypo
cg01514668	miR-129-2	11	8.5878E-13	7.42844E-07	12.95384	19.57678	−6.62294	0.58	0.46	0.70	Hypo
cg18576861	miR-3621	9	4.85176E-11	4.19677E-05	23.36382	30.90311	−7.53929	0.74	0.63	0.86	Hypo
cg20351875	miR-548C	12	1.0169E-10	8.79621E-05	16.31689	22.83412	−6.51723	0.67	0.55	0.79	Hypo
cg25147193	miR-181C	19	1.44145E-10	0.000124685	8.817696	13.97549	−5.157794	0.64	0.52	0.76	Hypo
cg11479035	miR-1276	15	2.1566E-10	0.000186546	84.82172	79.64312	5.1786	0.77	0.66	0.87	Hyper
cg06871184	miR-1238	19	4.3656E-10	0.000377624	64.27575	57.02732	7.24843	0.72	0.60	0.83	Hyper
cg13781167	miR-8081	9	1.58717E-09	0.001372905	64.99973	71.75161	−6.75188	0.70	0.58	0.81	Hypo
cg19756622	miR-518A1	19	3.09143E-09	0.002674085	80.68514	75.23586	5.44928	0.82	0.72	0.92	Hyper
cg17540499	miR-592	7	3.8092E-09	0.00329496	31.95645	39.38867	−7.43222	0.59	0.47	0.71	Hypo
cg13767940	miR-34B	11	1.06906E-08	0.009247326	11.87235	16.98866	−5.11631	0.64	0.52	0.76	Hypo
cg26438516	miR-1256	10	2.79234E-08	0.024153783	66.7636	60.35334	6.41026	0.71	0.60	0.83	Hyper

### Pathway analysis of significant miRNAs

**FIGURE 1:**
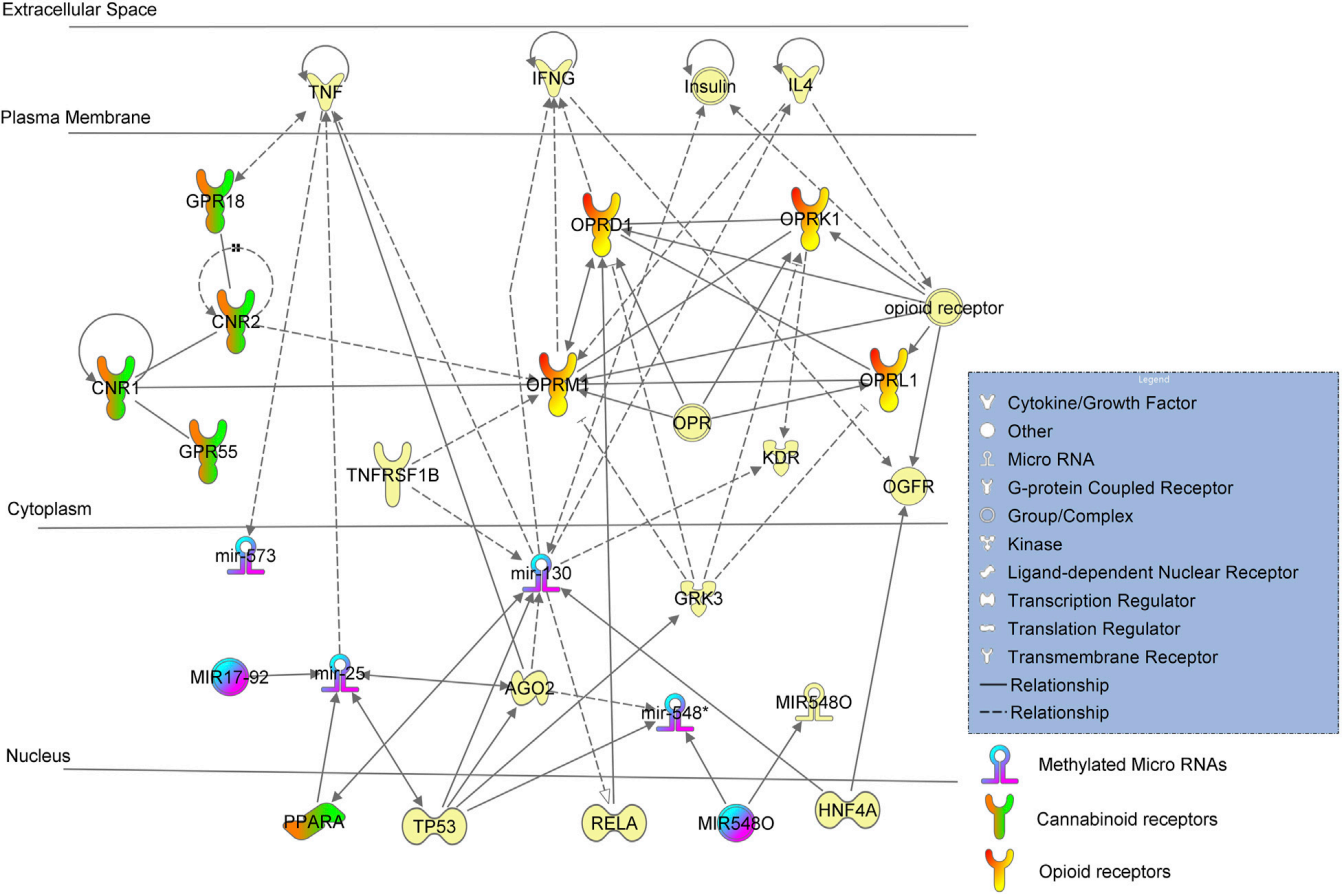
Ingenuity pathway analysis (IPA) of significant methylation regulators of miRNA-encoding genes and network analysis with a *p*-value <0.05 are depicted using + Opioids/+NOWS *versus* + Opioids/-NOWS.

**FIGURE 2:**
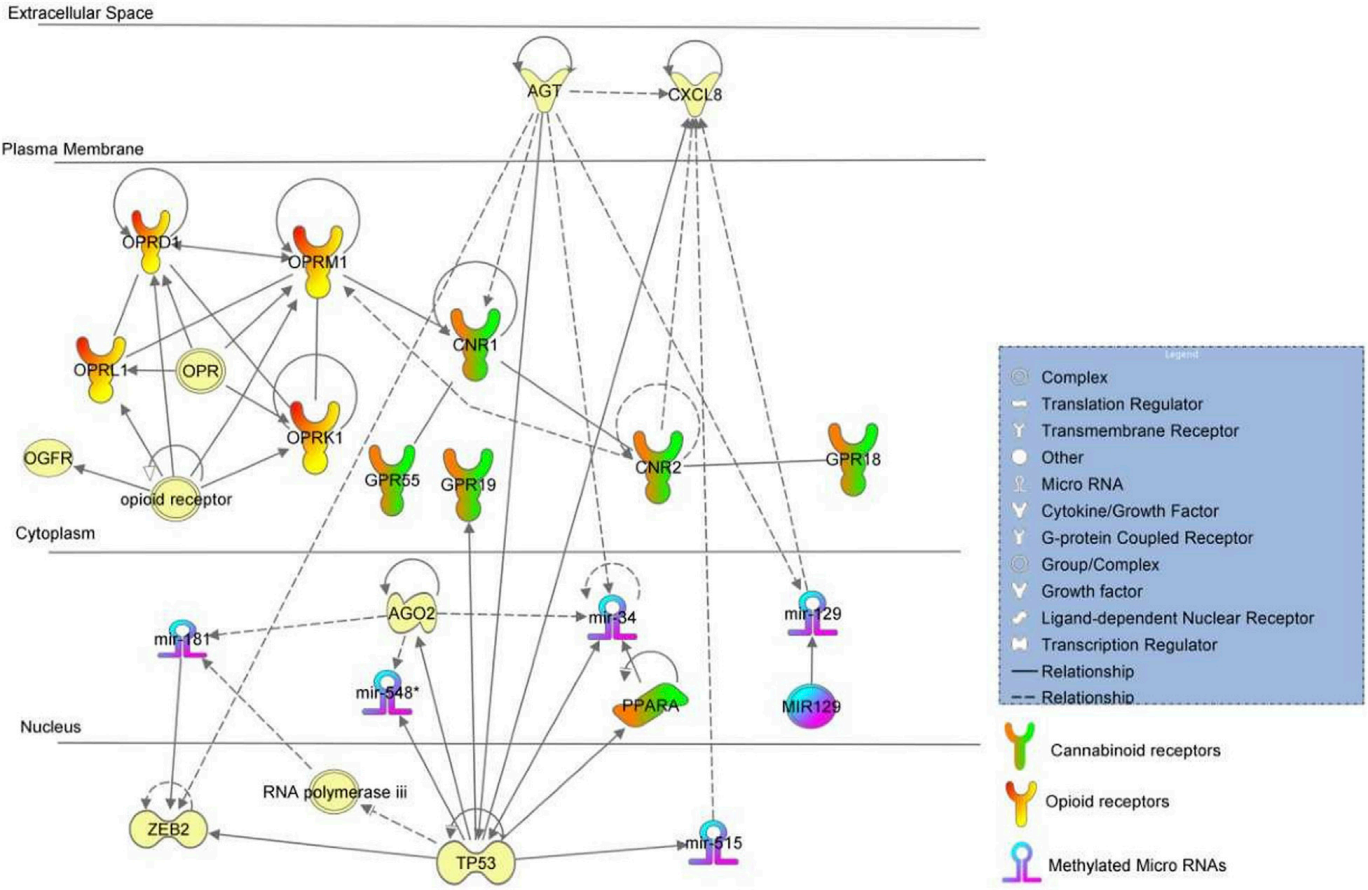
Pathway analysis of +Opioids/+NOWS and +Opioids/-NOWS *versus* -Opioids/-.

**FIGURE 3:**
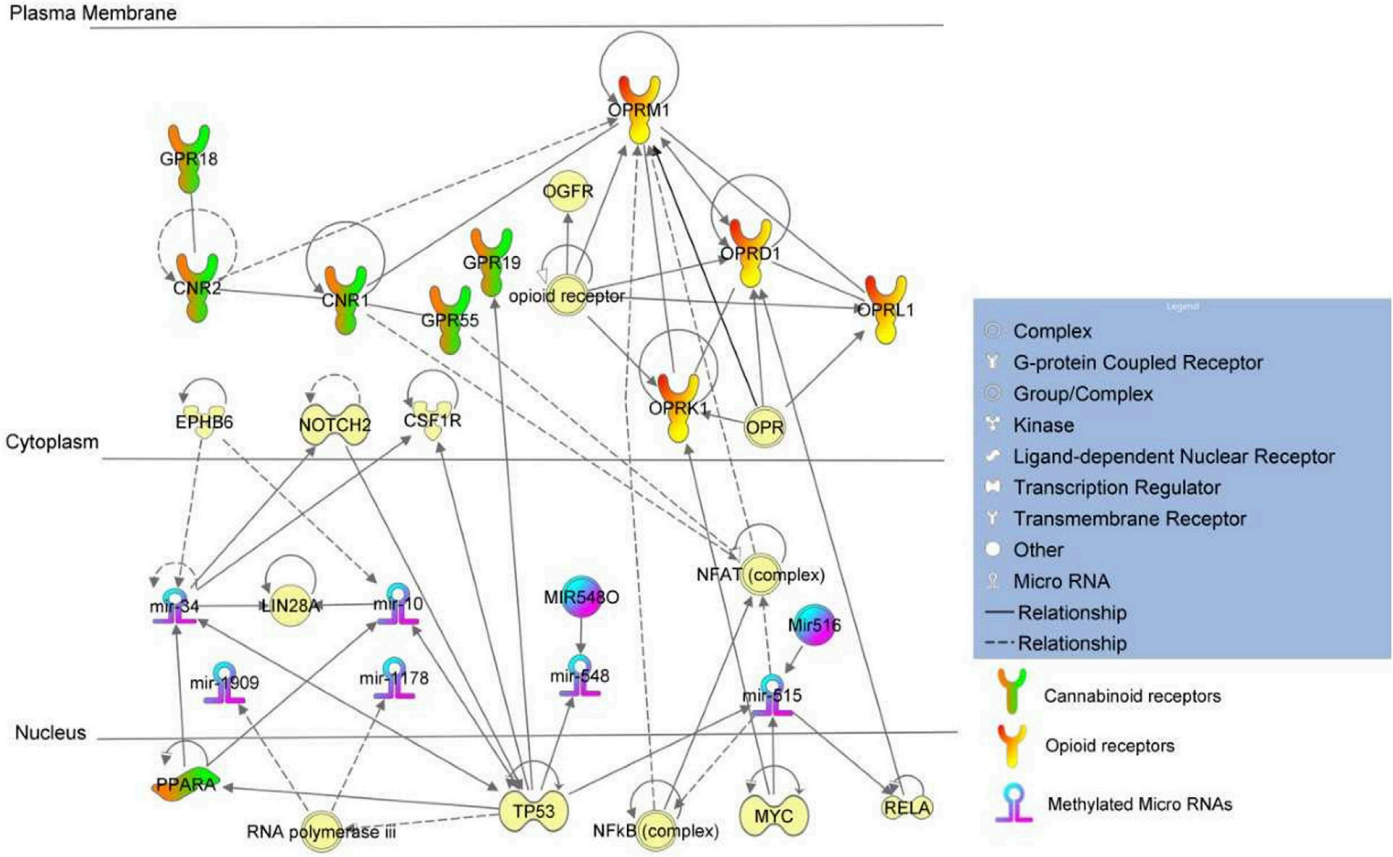
Pathway analysis of +Opioids/+NOWS *versus* -Opioids/-NOWS.

Gene ontology analysis found major dysregulated pathways affected by methylation changes on miRNAs associated with NOWS and OUD-related outcomes. A comparison was made between three groups i. Distinguishing NOWS from prenatal opioid-exposure without NOWS (+Opioids/+NOWS, *versus* + Opioids/-NOWS), ii. Distinguishing prenatal opioid use *versus* unexposed controls (OUD detection) (+Opioids/+NOWS and +Opioids/-NOWS *versus* -Opioids/-) and iii. distinguishing NOWS *versus* unexposed controls (+Opioids/+NOWS *versus* + Opioids/-NOWS). Significant functional biological processes regulated by these miRNAs were identified with the plausible molecular mechanism of differentially expressed miRNAs. Network interaction between miRNAs and their target genes was provided in Figure 1, Figure 2, and Figure 3.

### Target identification of differentially methylated miRNAs

MicroRNA Target Prediction and Functional Study Database (miRDB) server (http://miRdb.org) were used to predict putative targets of aberrantly methylated miRNAs. With a set target score of ≥60 (Wang, 2016), we searched the miRDB database for predictions of targets of some of these significant miRNAs. From the miRDB, we retrieved the target genes for 47 miRNAs. From this, we discovered multiple predicted targets for each miRNA (Adlakha and Saini, 2014). These predicted 47 miRNA targets were compared with the differentially methylated genes identified in our genome-wide methylation data of the same sample cohort (Radhakrishna et al., 2021b). The results showed that multiple predicted targets (genes) of these miRNAs were also found to be differentially methylated in our patients. Supplementary Tables S1 to S15 provide examples of miRNA targets discussed in the manuscript that are important and available in the miRDB database.

## Discussion

### Description of opioid use with NOWS vs. opioid use without NOWS

(opioid-exposed infants who required pharmacologic management for NOWS *versus* opioid-exposed infants that did not require treatment for NOWS) (+Opioids/+NOWS *versus* + Opioids/-NOWS) (Table 2). The *miR-130* family includes four members (*miR-130a, miR-130b, miR-301a, and miR-301b*) with the same source sequences and which perform similar biological functions (Wang et al., 2020). The *miR-130* mature miRNA is known to increase the expression of *PPARA* mRNA (Papi et al., 2013). *PPARA* is a nuclear hormone receptor that responds to certain types of cannabinoids (O'Sullivan, 2016).

*MiR-130* is regulated by *TP53, HNF4A, AGO2, TNFRFS1B,* and insulin, and was found to regulate the expressions of *KDR, IL4, IFNG, TNF,* and *RELA*. This regulation is likely dampened to a certain extent due to methylation, resulting in higher protein expressions and activations of the miRNA. *MiR-130* regulates *IL4*, which in turn regulates the mu-opioid receptor (MOR), whose principal ligands include opioid peptides and analgesics. The MOR also has an important role in dependence on other drugs of abuse, such as nicotine, cocaine, and alcohol via its modulation of the dopamine system.

Interestingly, *miR-130* putatively targets several other genes that are preferentially involved in brain disorders. For instance, *miR-130*, in combination with other miRNAs, jointly regulates *ATXN1*, which causes spinocerebellar ataxia type 1 (SCA1). SCA1 causes seizures, slurred speech, slowness of movement, and cognitive impairments. Similar neurological signs such as slurred speech or slowed movements are seen in OUD subjects, and conditions such as tremors and seizures are common in both OUD and infants with NOWS. Our genome-wide methylation analysis of NOWS revealed hypermethylation of the *ATXN1* gene (Radhakrishna et al., 2021b).

Inhibition of *miR-130* leads to decreased translocation of phosphorylated RELA protein to nuclei (Hazra et al., 2019). *RELA* protein (Chen et al., 2006) plays a significant role in the downstream activation of the *OPRD1* (also known as *DOR*, δ-opioid receptor, or delta-opioid receptor) is a paralog of *OPRM1* associated with opioid dependence gene that encodes the delta-opioid receptor (*DOR*) protein in humans. In the central nervous system, DOR and mu-opioid receptors (*MOR*) can interact and modulate each other’s and are involved in the regulation of pain (Beaudry et al., 2015; Olesen et al., 2018). *DOR* is responsible for reducing the intensity of pain signals, while *MOR* is responsible for reducing the frequency of pain signals. Heterodimerization of *MOR* and *DOR* can indeed impact the recruitment of beta-arrestin 2 (*ARRB2*) protein, which subsequently influences downstream intracellular signaling (Rozenfeld and Devi, 2007).

### Description of opioid exposure vs. unexposed controls

(opioid-exposed infants who required pharmacologic management for NOWS + opioid-exposed infants that did not require treatment for NOWS *versus* unexposed controls. (+Opioids/+NOWS plus + Opioids/-NOWS *versus* -Opioids/-NOWS). Table 3 describes the miRNA dataset from mothers with a history of opioid usage. This cohort was compared against normal unexposed controls and showed significant methylation differences in miR-34**,** miR-129, miR-181, and miR-548 among others.

The binding of the *AGO2* protein was found to occur with *miR-34* mature miRNAs (Wu et al., 2011). The presence of *AGT* proteins was found to increase expressions of mouse *miR-34* and *miR-129* mature miRNAs indicating positive regulation by *AGT* (Jin et al., 2012; Huo et al., 2019). *AGT* protein increases paracrine activation of *CNR1* protein (Turu et al., 2009), followed by clustering of *CNR1* with *CNR2*. Activated *CNR2* protein increases the expression of *OPRM1* (Borner et al., 2006) possibly resulting in an *OPRM1-OPRD1-OPRL1-OPRK1* feedback loop.

The binding of the *TP53* response element from the *miR-34* promoter and *TP53* protein was found to occur followed by targeting of *TP53* mRNA by *miR-34* mature miRNA. *TP53* protein further increases the transcription of the *miR-34* gene and *miR-34* mature miRNA was found to, in turn, upregulate the expression of acetylated (K382) *p53* protein followed by increasing activity of *p53* protein (Feng et al., 2011). Protein modification of *AGO2* leads to an increased expression of *miR-548* and *miR-181* (Jin et al., 2012; Garibaldi et al., 2016). Homozygous experimental *p53* gene deletion was found to decrease the expression of *miR-548* mature miRNA (Shin et al., 2009). Experimental interference of human *p53* mRNA by shRNA led to an increased expression of *miR-519* mature miRNA (Garibaldi et al., 2016). Binding of 3′UTR from *ZEB2* mRNA and *miR-181A* mature miRNA occurs, indicating probable regulation of *ZEB2* mRNA. Negative regulation of *TP53* in this pathway leads to activations of several genes in the pathway including *PPARA* which is a nuclear hormone receptor capable of taking certain cannabinoid ligands.

Women who used illicit or unprescribed opioids during pregnancy have a higher risk of fetal growth restriction and preterm delivery (Maghsoudlou et al., 2017). Recent studies have shown differential *miR-548* expression profile was associated with spontaneous preterm births (Gray et al., 2017). Placental DNA methylation of *miR-548* and *WWTR1* genes influence insulin sensitivity during pregnancy, and preterm birth and is linked to insulin resistance (Hofman et al., 2004; Mathai et al., 2012). Interestingly, nine of the 47 dysregulated miRNAs belonged to the *miR-548* family, including *miR548G, miR548F1, miR548W, miR548H3, miR548AE2, miR548F5, miR548F3, miR548C*, and *miR548C*, all of which were hypermethylated. Moreover, *WWTR1* is also hypermethylated in the present study.

### NOWS vs. unexposed controls

(opioid-exposed infants who required pharmacologic management for NOWS *versus* unexposed controls (+Opioids/+NOWS *versus* -opioids/-NOWS, control) (Table 4). MiR-515 is the largest miRNA gene cluster in humans (Zhang M. et al., 2016), the family members of *miR-515* include *miR-516* and *miR-518* (Zhang M. et al., 2016). *MiRNA-515* and *miR-34* are found to be connected directly to opioid and cannabinoid receptors respectively. *MiR-515* was found to regulate opioid genes *OPRM1*, and *OPRD1*, while miR-34 was found to regulate cannabinoid genes *GPR19* and *PPARA* via Notch and *TP53* proteins.

Mature miR-515 is known to decrease the activity of the human nuclear factor kappa B (NFkB) complex (Keklikoglou et al., 2012), however, in the absence of active *miR-515* due to methylation as seen in the current cohort, the Nfkb complex is actively expressed leading to continued expression of *OPRM1* mRNA. Thus, methylation of *miR-515* results in the transcription of human *OPRM1* mRNA which otherwise would be under the strict regulation of the *NFkB* complex (Kraus et al., 2003; Memet, 2006).

*RELA* mRNA is targeted by *miR-515* mature miRNA leading to its decreased expression (Keklikoglou et al., 2012). Interference of *Rela* mRNA by siRNA decreases the expression of *Oprd1* mRNA. *RELA* protein increases activation of the reporter gene with a promoter fragment (-262–1) from the *OPRD1* gene (Chen et al., 2006). Methylation of miR-515 leads to the absence of regulation leading to an increased expression of *RELA*. Increased *RELA* subsequently increases its downstream partner *OPRD1*. The *OPRM1* and *OPRD1* genes were also found to be distinctively methylated in the previously described datasets. *OPRM1* was found hypomethylated in mothers who used opioids during pregnancy and delivered NOWS newborns which required immediate medical care when compared to mothers who had used opioids during pregnancy and delivered newborns without NOWS. In contrast, *OPRD1* was found hypermethylated in mothers with a history of opioid usage who delivered babies without NOWS when compared against unexposed controls. Both *OPRD1* and *OPRM1* were found to be distinctly methylated in the current dataset of mothers who used opioids and delivered NOWS babies when compared against unexposed controls. However, *PPARA* activation status differed across these three datasets.

### Role of *PPARA* in conjunction with opioid and cannabinoid receptors

The absence of active *miR-130* in the +Opioid/+NOWS group may have led to decreased expression of *PPARA* mRNA, therefore, leading to reduced production of oxidation enzymes to effectively scavenge for free radicals generated by oxidative stress from opioid use. This may have resulted in mothers delivering newborns with NOWS that required medical intervention. Interestingly, when the Opioid+/NOWS- group was compared against unexposed controls, *PPARA* was found to be under the positive regulation of *TP53* leading to its activation. This protective role of *PPARA* was absent in the +Opioids/+NOWS cohort of mothers when compared against unexposed controls. This is possibly due to the active status of *miR-134* and/or to a different set of regulatory partners Notch and *TP53* whose role in this context is not known and can only be speculated. MiR-34 was found to regulate cannabinoid genes including *PPARA* via Notch and *TP53* proteins. Though the anti-oxidation activity of *PPARA* was probably negated by the extensive and consistent activations of opioid genes *OPRM1,* and *OPRD1*, unlike seen in any of the other two cohorts and thus resulting in mothers delivering NOWS newborns that required medical care. Moreover, *miR-34* has a functional role in the regulation of stress-induced anxiety, depression with suicidal ideation (Haramati et al., 2011). Anxiety and depression are common and normal phenomena, in mothers of infants experiencing NOWS (Corr et al., 2020).

*SIRT1* which is hypomethylated in the current cohort study (FDR) *p* ≤ 0.05) (Radhakrishna et al., 2021b), is a nicotinamide adenosine dinucleotide (NAD)-dependent deacetylase that removes acetyl groups from various proteins. *SIRT1* normally functions to limit the expression of *miR-134*, which targets the critical activity-dependent transcription factor (CREB) essential for learning and memory (Lv et al., 2013; O'Neal-Moffitt et al., 2014; Liu et al., 2017). *SIRT1* exerts neuroprotection against ischemic injury and various neurodegenerative disorders (Xu et al., 2018). Dysregulation of brain *SIRT1* activity can have devastating brain consequences including neurological dysfunction (Xu et al., 2018).

Experimental interference of human *p53* mRNA by shRNA led to an increased expression of *miR-519* mature miRNA (Garibaldi et al., 2016). Homozygous experimental *p53* gene deletion was found to decrease the expression of *miR-548* mature miRNA (Shin et al., 2009). *MiR-10* which belongs to the *miR-125A* family member (Tehler et al., 2011) was found to decrease the activation of *p53* (Joo et al., 2013). Studies on mouse mmu-miR-10 mature miRNA were found to decrease the expression of mouse *p53* protein (Sen et al., 2014). Targeting of *LIN28* mRNA by *miR-10* and *miR-34* mature miRNAs was found to occur leading to decreased translation of *LIN28* (Zhong et al., 2010; Jain et al., 2012). *p53* (*TP53*) protein was found to decrease the binding of *BRF1* protein and RNA polymerase iii complexes (Crighton et al., 2003). Experimental inhibition of active RNA polymerase iii complexes was found to result in increased expression of *miR-1909* while decreasing the expression of *miR-1178* mature miRNA (Koo et al., 2015) demonstrating the role of transcriptional regulation by RNA polymerase iii.

### Limitations

The study has some limitations, namely, a lack of racial or ethnic diversity, given that it focused on individuals of European origin. A second limitation is that we have not replicated our findings in an independent study cohort, and therefore, we will continue to carry out *in vitro* and *in vivo* experiments to validate the conclusions in the future.

### Summary and future directions

To our knowledge, this is the first report about miRNA methylation levels of NOWS on a previously well-documented large cohort of placental tissue specimens with genome-wide methylation profiles and gene expression profiles. NOWS is associated with an altered miRNA methylation pattern in the placenta, suggesting that miRNA deregulation is involved in the pathogenesis of NOWS. The current experimental data suggest that epigenetic variations in the placental tissue can serve as surrogate markers for brain health at birth, and thus infant micro-RNA “signatures” can predict the severity of NOWS even before withdrawal symptoms begin. The differential methylation of several miRNAs has been found in association with NOWS. The findings have biological plausibility, and they include several known biological pathways and genes with possible mechanisms associated with NOWS development. The identified dysregulated pathways and genes may therefore provide important opportunities for the development of novel future miRNA-based therapeutic approaches for NOWS. Several preclinical and clinical trials have been reported for miRNA-based therapeutics and few reports indicate that miRNA expression levels can be modified by modulating the miRNA processing pathway. However, validation with large NOWS cohorts and using robust techniques for DNA methylation analysis of miRNA genes are warranted before the application in clinical practice.

## Data Availability

The original contributions presented in the study are included in the article/Supplementary Material, further inquiries can be directed to the corresponding author.

## Abbreviations

Hypo, Hypomethylation; Hyper; Hypermethylation.
